# Celecoxib inhibits NLRP1 inflammasome pathway in MDA-MB-231 Cells

**DOI:** 10.1007/s00210-024-03286-2

**Published:** 2024-07-11

**Authors:** Ege Arzuk, Derviş Birim, Güliz Armağan

**Affiliations:** 1 Department of Pharmaceutical Toxicology, Faculty of Pharmacy, Ege University, Bornova, 35040 Izmir, Turkey; 2 Department of Biochemistry, Faculty of Pharmacy, Ege University, Bornova, Izmir, Turkey

**Keywords:** NLRP1 inflammasome, Anticancer drug, Triple-negative breast cancer, Celecoxib, COX-2

## Abstract

NLRP1 is predominantly overexpressed in breast cancer tissue, and the evaluated activation of NLRP1 inflammasome is associated with tumor growth, angiogenesis, and metastasis. Therefore, targeting NLRP1 activation could be a crucial strategy in anticancer therapy. In this study, we investigated the hypothesis that NLRP1 pathway may contribute to the cytotoxic effects of celecoxib and nimesulide in MDA-MB-231 cells. First of all, IC_50_ values and inhibitory effects on the colony-forming ability of drugs were evaluated in cells. Then, the alterations in the expression levels of NLRP1 inflammasome components induced by drugs were investigated. Subsequently, the release of inflammatory cytokine IL-1β and the activity of caspase-1 in drug-treated cells were measured. According to our results, celecoxib and nimesulide selectively inhibited the viability of MDA-MB-231 cells. These drugs remarkably inhibited the colony-forming ability of cells. The expression levels of NLRP1 inflammasome components decreased in celecoxib-treated cells, accompanied by decreased caspase-1 activity and IL-1β release. In contrast, nimesulide treatment led to the upregulation of the related protein expressions with unchanged caspase-1 activity and increased IL-1β secretion. Our results indicated that the NLRP1 inflammasome pathway might contribute to the antiproliferative effects of celecoxib in MDA-MB-231 cells but is not a crucial mechanism for nimesulide.

## Introduction

Breast cancer remains a significant cause of mortality among women worldwide, with a consistently increasing incidence rate. Significant advancements in anticancer treatments have notably improved outcomes for patients. However, difficulties must be overcome to enhance therapeutic effectiveness and prevent adverse effects, particularly in triple-negative breast cancer (Carey et al. 2006). Therefore, it is crucial to present effective therapeutic strategies by searching for novel pharmacological mechanisms and targets, including new drugs and repurposing existing ones (Masoud and Pagès 2017).

Inflammasomes are crucial components of the innate immune system and have a critical function in protecting organisms against cellular stress, infections, and tissue damage. However, numerous studies have revealed that chronic inflammation and inflammasomes are notably associated with the development of various diseases, including cancer, neurodegenerative disorders, obesity, diabetes, atherosclerosis, and ulcerative colitis (Strowig et al. 2012). Recently, there has been a growing interest in investigating the potential contributions of inflammasomes to tumor development, progression, and metastasis (Karki and Kanneganti 2019). Interestingly, the role of inflammasomes in tumorigenesis and carcinogenesis is still controversial due to their tumor-promoting or suppressing effects, depending on the type of inflammasome and cancer (Thi and Hong 2017). Inflammasome-derived components may protect against colitis-associated colon cancer (Sharma and Kanneganti 2023). However, various reports indicate that inflammasomes may promote tumor growth in breast, lung, and skin cancer (Kantono and Guo 2017).

In breast cancer, the excessive activation of certain types of inflammasomes is associated with carcinogenesis, metastasis, and recurrence (Guo et al. 2016). In particular, the contribution of the NLRP1 inflammasome pathway to the development and progression of breast cancer is noteworthy (Wei et al. 2017). The activation of NLRP1 inflammasome, consisting of NLRP1, ASC adapter protein, and pro-caspase-1 effector protein, results in caspase-1 activation. Upon activation, caspase-1 triggers the release of IL-1β and IL-18, leading to pyroptosis in the cell (Taabazuing et al. 2020). In breast cancer tissues, expression levels of NLRP1 are substantially upregulated (Guo et al. 2016). The overexpression of NLRP1 and increased levels of IL-1β in the tumor microenvironment have been emphasized to promote breast cancer malignity, progression, and migration (Elaraj et al. 2006; Wei et al. 2017). Therefore, targeting the NLRP1 inflammasome pathway represents a novel therapeutic approach.

It has been reported for over 20 years that COX inhibitors, specifically COX-2 selective inhibitors, may have anticancer properties. COX-2 is frequently overexpressed in various human malignant tumors, including breast cancer, similar to NLRP1 (Díaz-Cruz et al. 2005). The increased COX-2 expression level is associated with breast tumor growth rate and malignancy, as well as hyperproliferation, angiogenesis, and metastasis (Costa et al. 2002; Liu et al. 2001). Celecoxib, a potent COX-2 enzyme inhibitor, and nimesulide, a preferential COX-2 inhibitor, are frequently prescribed due to their analgesic and anti-inflammatory properties (Bennett and Villa 2000; Derry and Moore 2013). It has been shown that celecoxib suppresses cancer cell proliferation and reduces cancer incidence by numerous preclinical, clinical, and epidemiological studies (Ashok et al. 2011; Lanza-Jacoby et al. 2013; Woditschka et al. 2008). Previous investigations provide compelling results that also nimesulide could suppress tumor growth (Eibl et al. 2003; Hida et al. 2000; Li et al. 2003; Pan et al. 2003). Several COX-2-dependent or -independent mechanisms have been proposed for the antiproliferative effects of celecoxib and nimesulide (Chen et al. 2009; Li et al. 2018; Nakatsugi et al. 2000; Shaik et al. 2004; Wen et al. 2020); however, the current understanding of the precise mechanisms underlying the anticancer potential of relevant drugs is limited. The expression of COX-2 can be induced by inflammatory cytokines released due to inflammasome activation (Kuwano et al. 2004). Additionally, suppressing COX-2 enzyme activity or expression reduces NLRP3 inflammasome activation, serum IL-1β, and caspase-1 activity in cells (Hua et al. 2015). Besides NLRP3, COX-2 inhibitors may have anticancer effects by inhibiting the inflammatory activation of NLRP1, which is predominantly highly expressed in breast cancer tissue. However, comprehensive studies investigating the role of NLRP1 activation in the antiproliferative effects of COX-2 inhibitors in breast cancer are limited.

In the present study, we hypothesized that the NLRP1 inflammasome pathway may be involved in the antiproliferative and cytotoxic effects of celecoxib in the MDA-MB-231 cells. Nimesulide was included in the study to compare its results with celecoxib, which has higher COX-2 selectivity. We first determined cytotoxic effects of drugs via WST-1 and colony formation assay. Then, we investigated alterations induced by celecoxib or nimesulide in the expression levels of NLRP1 inflammasome components. Finally, we measured the caspase-1 activity and IL-1β secretion of each drug-treated cell.

## Materials and methods

### Cell culture and materials

MDA-MB-231, a triple-negative breast cancer cell line (ATTC HTB-26, USA), MCF-7, the estrogen receptor (ER)-positive human breast cancer cell line (ATTC HTB-22, USA), SK-BR-3, human epidermal growth factor receptor 2 (HER2)-positive breast cancer cell line (ATTC HTB-30, USA), and MCF-10A, a non-tumorigenic epithelial cell line (ATTC CRL-10317, USA), were maintained at 37 °C in a 5% CO_2_ atmosphere. Breast cancer cells were cultured in DMEM supplemented with 10% FBS and 1% penicillin/streptomycin. MCF-10A cell line was cultured in Lonza Mammary Epithelial Cell Medium (Lonza/Clonetics, USA) enriched with MEGMTM SingleQuot Kit (Lonza). The cells were regularly monitored with the inverted microscope, and subculturing protocols were applied when they reached 80% confluence. Anti-NLRP1, anti-cleaved caspase-1, anti-ASC, and anti-β-actin antibodies were obtained from the Cell Signaling (Ann Arbor, MI). The COX activity kit (MAK414) and IL-1β ELISA kit (RAB0273) were acquired from Sigma-Aldrich (St. Louis, MO), and caspase-1 activity kit (ab39412) was purchased from Abcam (MA, USA). Celecoxib and nimesulide were kindly provided by Deva Pharmaceutical Company (Turkey). All other chemicals and materials were obtained from Sigma-Aldrich (St. Louis, MO).

### Preparation of drug solutions

Celecoxib and nimesulide were dissolved in DMSO to prepare leading stock solutions of 10 mM. Then, stock solutions were diluted with the appropriate volume of medium, and cells were incubated at the desired final concentrations of drugs. The maximum DMSO concentration did not exceed 0.1% (v/v).

### Determination of cytotoxicity of drugs by WST-1 assay

MDA-MB-231 and MCF-10A cells (two independent cultures) were seeded into 96-well plates (0.6 × 10^4^ cells/well). Following overnight, the cells were treated with increasing concentrations of each drug (0–100 µM for MDA-MB-231 cells and 0–400 µM for MCF-10A cells) for 48 h. The cells exposed to 0.1% DMSO were used as a control. At the end of the incubation, the WST-1 assay protocol was performed in accordance with the previous studies (Jia et al. 2014). Untreated and treated cells were incubated with the 10 µl WST-1 reagent for 3 h at 37 °C. The absorbance was measured using a Varioskan microplate reader at 430 nm. The cell viability was calculated as a percentage of the untreated control. IC_50_ values were calculated using GraphPad Version. Fifty percent, 75%, and 25% inhibitory concentration (IC_50_, IC_75_, and IC_25_) values were calculated using GraphPad Prism software 8.4 (GraphPad, San Diego, CA). Selectivity indices (SI) were calculated from the IC_50_ ratio of MCF-10A to MDA-MB-231 cells.

### Colony formation assay

The colony formation assay determines an agent’s long-term cytotoxic effects by measuring a single cell’s colony formation ability (Elangovan et al. 2008). MDA-MB-231 cells were seeded in a 6-well plate at 500 cells/well and allowed to attach for 24 h. Then, each drug was added into wells at three doses (IC_25_, IC_50_, and IC_75_). Following the incubation period (14 days), the medium was removed, and the wells were washed with PBS. Cells were fixed using 70% ethanol. Subsequently, the wells were incubated with 1.25% crystal violet for 1 h. Finally, the stained colonies were counted using an inverted microscope, and the number of colonies was compared with the control (% 0.1 DMSO) (Elangovan et al. 2008).

### Western blotting

Initially, a western blot analysis was conducted to compare the levels of NLRP1 protein in various breast cancer cell lines with that of MCF-10A, a healthy cell line. Then, the effects of drugs on the expression levels of NLRP1, ASC, and cleaved caspase-1 proteins in MDA-MB-231 cells were investigated. Cells (3 × 10^5^ cells/well) were treated with drugs at IC_50_ concentration for 48 h. After incubation, the cells were harvested and lysed with RIPA buffer containing a 1% protease inhibitor cocktail. The protein contents were measured by Bradford agent (Bradford 1976). Cellular proteins (30 µg) were separated with SDS-PAGE at 120 V and transferred to PVDF membranes at 100 mV for 2 h at + 4 °C. Membranes were blocked with 5% milk powder for 1 h. Then, membranes were incubated with anti-NLRP1, anti-cleaved-caspase-1, anti-ASC, anti-cleaved-caspase-1, or anti-β-actin, followed by incubation with secondary antibodies. Vilber Lourmat Fusion FX7 system was used to reveal the protein bands through enhanced chemiluminescence. Relative protein levels were normalized to β-actin (Zhai et al. 2017).

### IL-1β release

MDA-MB-231 cells were treated with different concentrations of drugs (IC_25_, IC_50_, and IC_75_) for 48 h. The levels of IL-1β released into the medium were measured using an ELISA assay following the commercial kit protocol. For this purpose, at the end of the incubation period, the supernatant in the wells was collected and applied to wells coated with a monoclonal antibody for IL-1β. Biotinylated anti-IL-1β was added to the standards and samples, followed by 3 h of incubation at room temperature. Subsequently, the wells were washed, streptavidin HRP was added, and the second incubation step was started for 30 min at room temperature. Then, the wells were washed, and chromogen TMB was added. After 20 min, stopper solution was added, and the absorbance was measured at 450 nm by a microplate reader. The intensity of absorbance directly reflects the concentration of IL-1β (Escobar et al. 2015).

### Statistical analyses

Experiments were repeated three times and performed in triplicate. Data was expressed as mean ± SD. Kolmogorov–Smirnov was performed as a normality test. Statistical differences were analyzed using one-way ANOVA, following Dunnett’s post hoc test. *p* < 0.05 was considered statistically significant.

## Results

### Cytotoxic effects of celecoxib and nimesulide on MDA-MB-231

**Fig. 1:**
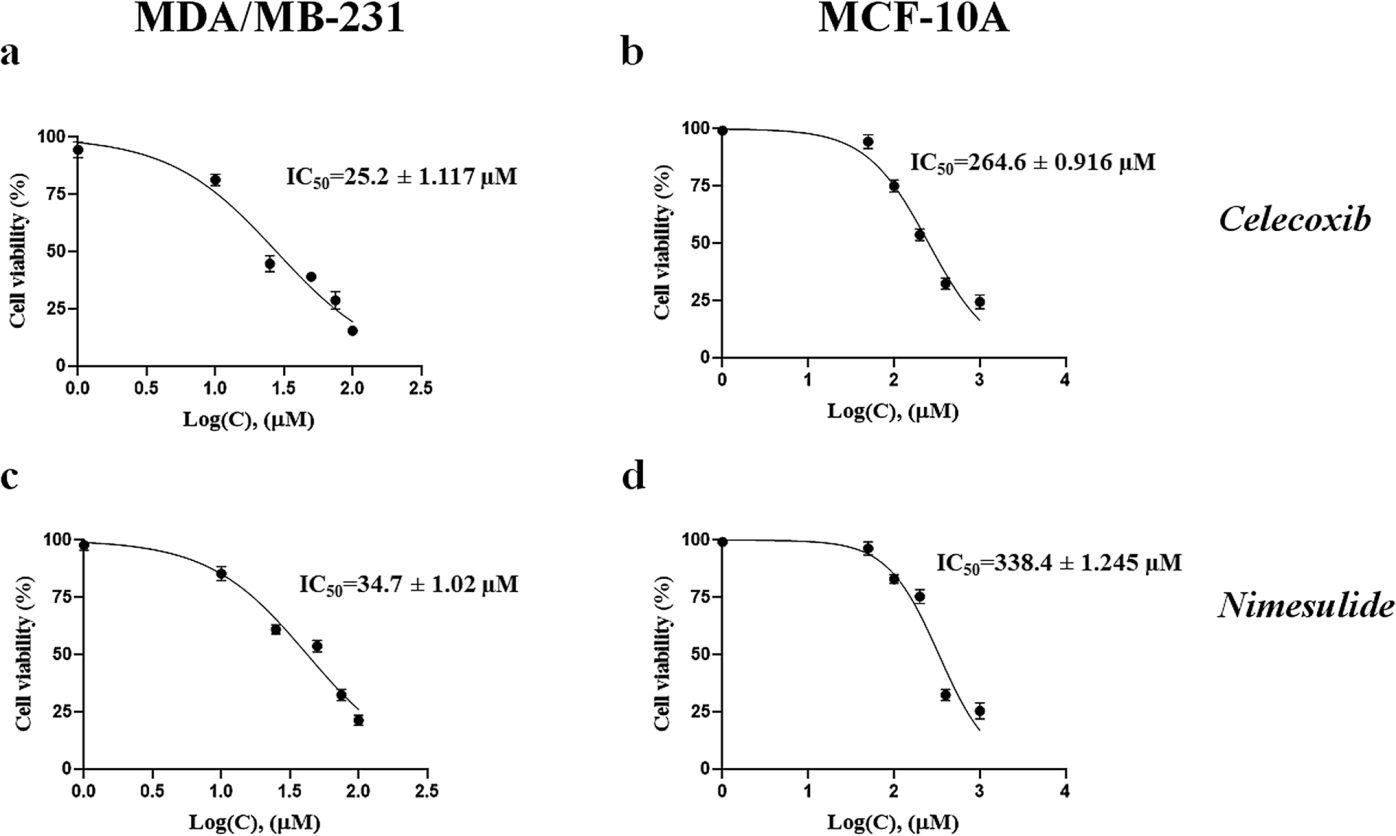
Cell viability analysis after incubation with celecoxib or nimesulide. MDA-MB 231 (**a**, **c**) and MCF-10A (**b**, **d**) cells were treated with increasing concentrations of each drug for 48 h, and the cell viability was measured by WST-1 assay. **a** Celecoxib-MDA-MB-231 cells; **b** celecoxib-MCF-10A cells; **c** nimesulide-MDA-MB-231 cells; **d** nimesulide-MCF-10A cells. Each value shown as mean ± S.D. was expressed as a percentage of the control (0.1% DMSO), and IC_50_ values were calculated from the dose–response curves using GraphPad Prism® 8.4.2 software. 1% Triton-X was used as a positive control (data not shown)

The cell viability assay was performed to assess the cytotoxic potential of each drug and determine the working concentrations for further studies. Hence, MDA-MB-231 and MCF-10A cells were treated with increasing concentrations of celecoxib or nimesulide, and IC_25_, IC_50_, and IC_75_ values were calculated using GraphPad (Table 1). Both drugs exhibited dose-dependent cytotoxicity on MDA-MB-231 cells; however, they were non-toxic to MCF-10A cells at dose levels below 200 µM. As shown in Fig. 1, celecoxib and nimesulide inhibited cell viability of MDA-MB-231 cells with IC_50_ values of 25.2 ± 1.117 µM and 34.7 ± 1.02 µM, respectively (Fig. 1a, c).

**Table 1:** Drug-induced cytotoxicity in MDA-MB-231 and MCF-10A cells. Cell viability was assessed using the WST-1 assay, and the IC_25_, IC_50_, and IC_75_ values were determined. Additionally, the selectivity index (SI) was determined by dividing the IC_50_ of MCF-10A by that of MDA-MB-231

Drugs	IC25 (µM)	IC75 (µM)	IC50 (µM)		Selectivity index (SI)
	MDA-MB-231	MDA-MB-231	MDA-MB-231	MCF-10A	
Celecoxib	10.19 ± 1.038	72.8 ± 1.124	25.2 ± 1.117	264.6 ± 0.916	10.5
Nimesulide	18.25 ± 1.001	93.28 ± 1.105	34.7 ± 1.02	338.4 ± 1.245	9.8

These drugs exhibited selective cytotoxicity towards breast cancer cells, as supported by the significantly (*p* < 0.05) higher IC_50_ values of MCF-10A (celecoxib; 264.6 ± 0.916 µM and nimesulide; 338.4 ± 1.245 µM) compared to MDA-MB-231 cells. The SI ratios of celecoxib and nimesulide were 10.02 and 9.8, respectively (Table 1). In addition to IC_50_, we calculated the IC_25_ and IC_75_ values of each drug in MDA-MB-231 cells to perform assays under slight or high cytotoxicity conditions and investigate dose dependency (Table 1).

### Celecoxib and nimesulide decreased the colony formation potentials of MDA-MB-231 cells

**Fig. 2:**
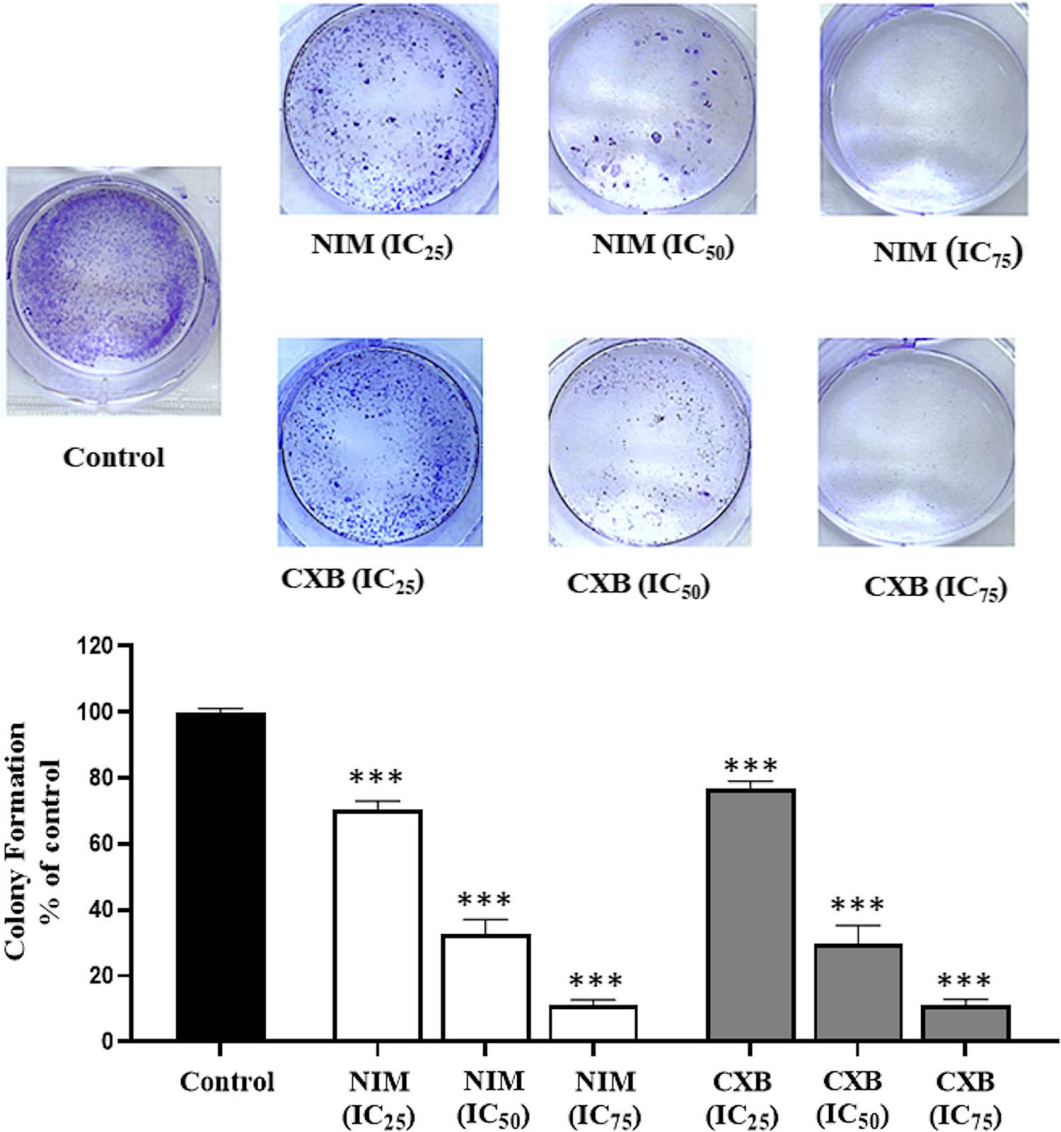
Celecoxib and nimesulide inhibit the colony formation ability of MDA-MB-231 cells. Cells were treated with each drug at three dose levels (IC_25_, IC_50_, and IC_75_) for 14 days. Following the treatment period, colonies were stained by 1.25% crystal violet. NIM, nimesulide; CXB, celecoxib. The number of surviving colonies was expressed as a percentage of the control (0.1% DMSO). Values were expressed as mean ± S.D. ***p < 0.0001 (vs. control group)

The colony-forming ability of MDA-MB-231 cells was evaluated following treatment with celecoxib or nimesulide to observe the long-term effects of the compounds. For this purpose, cells were exposed to each drug for 14 days at three dose levels (IC_25_, IC_50_, and IC_75_). At the end of the period, the number of colonies was normalized to the control. As shown in Fig. 2, both drugs significantly reduced the proliferation rate of MDA-MB-231 cells. The number of colonies decreased by 29% and 67% with celecoxib concentrations of IC_25_ and IC_50_, respectively. Treatment with nimesulide at IC_25_ and IC_50_ concentrations resulted in a 22% and 67.3% reduction in colony numbers, respectively. It was observed that exposure to IC_75_ doses of drugs almost wholly inhibited the colony-forming ability of cells.

### Western blot analyses

**Fig. 3:**
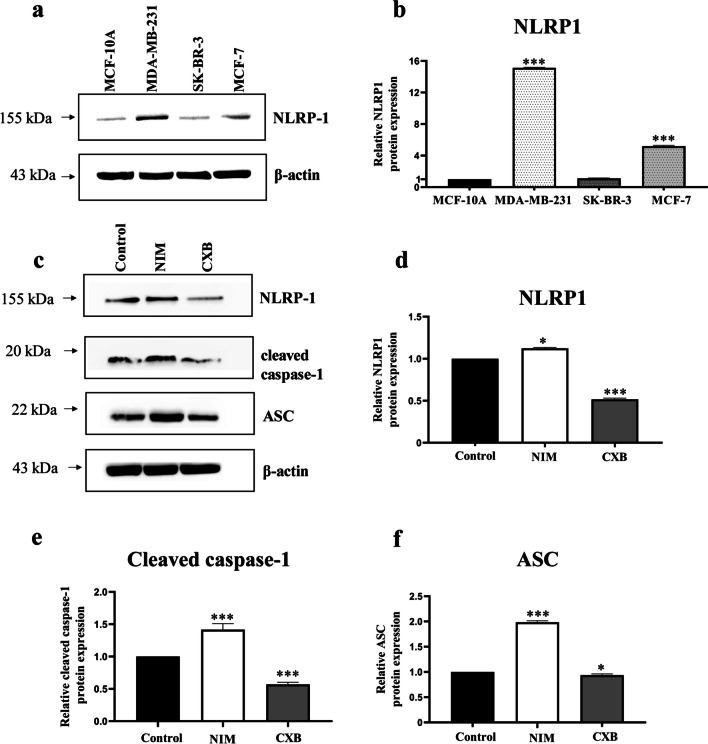
Protein expression of NLRP1, cleaved-caspase-1, ASC, and β-actin. **a** Semi-quantitative bands probed with anti-NLRP1, and -β-actin antibodies in several breast cell lines; **b** densitometric analyses of NLRP1 protein expression in several breast cell lines; **c** semi-quantitative bands probed with anti-NLRP1, -cleaved caspase-1, -ASC, and -β-actin antibodies in MDA-MB-231 cells; **d** densitometric analyses of NLRP1 protein expression in MDA-MB-231 cells; **e** densitometric analyses of cleaved caspase-1 protein expression in MDA-MB-231 cells; **f** densitometric analyses of ASC protein expression in MDA-MB-231 cells. Band density was measured with ImageJ software. The protein values were normalized to β-actin. NIM, nimesulide; CXB, celecoxib. For **b**; ***p < 0.0001 compared with MCF-10A, for Fig. 3d–f; *p < 0.05, ***p < 0.0001 compared with the control group (0.1% DMSO)

The results from the western blot analysis revealed that the level of NLRP1 protein expression was significantly upregulated in MDA-MB-231 cells (Fig. 3a) compared to the healthy cell line. Indeed, the NLRP1 protein expression in MDA-MB-231 cells was found to be approximately 15.1 times higher than in MCF-10A cells (Fig. 3b). When other breast cancer cell lines were examined, it was observed that SK-BR-3 cells expressed NLRP1 protein in almost similar amounts as healthy breast epithelial cells. In addition, the expression level of NLRP1 in MCF-7 cells was around 5.2 times higher than in MCF-10 cells (Fig. 3b). The significant upregulation of NLRP1 protein levels in MDA-MB-231 cells suggests that the NLRP1 inflammasome pathway may be a potential therapeutic target for triple-negative breast cancer. Therefore, we investigated whether the NLRP1 inflammasome signaling pathway plays a role in the antiproliferative effects of these drugs in MDA-MB-231 cells. First, we determined the respective effects of both drugs on the expression of NLRP1 inflammasome components: NLRP1, ASC, and cleaved-caspase-1. For this purpose, the cells were incubated with each drug for 48 h, and then western blot analysis was performed.

The expression of NLRP1 was remarkably downregulated by celecoxib at IC_50_ concentration after 48 h. Celecoxib induced about 48.4% reduction of NLRP1 protein expression in MDA-MB-231 cells (Fig. 3d). Additionally, the drug significantly decreased the expression of cleaved caspase-1 and ASC, which are considered other significant components of the NLRP1 inflammasome (Fig. 3e, f). Celecoxib reduced caspase-1 expression by 43.2% and ASC expression by 6.3%. Surprisingly, the expression levels of all three proteins were upregulated by nimesulide (IC_50_). Treatment with nimesulide led to a 12.4% increase in NLRP1 expression, a 41.8% increase in caspase-1 expression, and a 98% increase in ASC expression (Fig. 3). Our findings indicate that celecoxib reduces the expression levels of NLRP1 inflammasomal components and may inhibit its activation. However, the findings of nimesulide were unexpected.

### Effects of celecoxib and nimesulide on caspase-1 enzyme activity

**Fig. 4:**
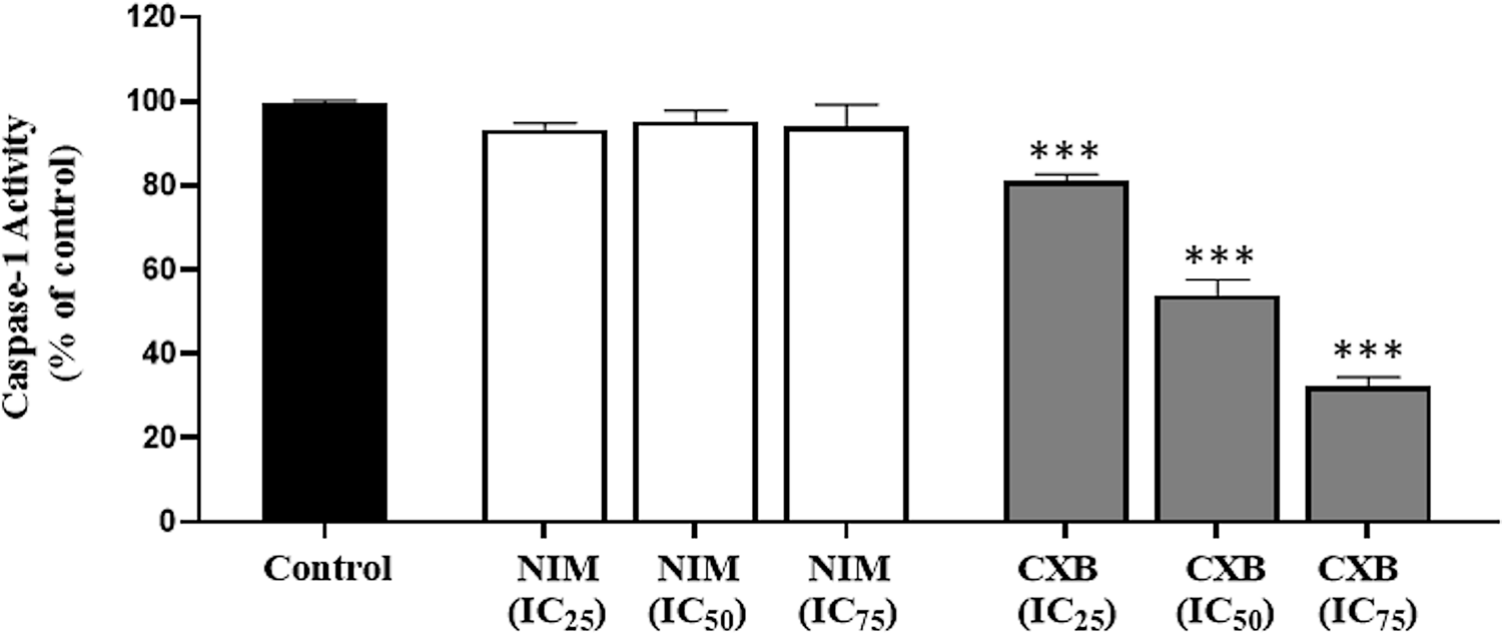
The effects of celecoxib and nimesulide on caspase-1 activity. MDA-MB-231 cells were treated with different concentrations of each drug for 48 h. Caspase-1 activity was determined fluorometrically. NIM, nimesulide; CXB, celecoxib. 0.1% DSMO was used as a solvent control. Values were represented as mean ± S.D. ***p < 0.0001, compared to control

Figure 4 demonstrates the caspase-1 activities of MDA-MB-231 cells treated with increasing concentrations of celecoxib or nimesulide for 48 h. Based on the results, the activity of caspase-1 was dramatically inhibited by celecoxib compared to control cells. Celecoxib at IC_25_, IC_50_, and IC_75_ concentrations reduced enzyme activity by 19%, 46%, and 68%, respectively. The inhibition of caspase-1 activity was associated with lower protein expression of NLRP1, cleaved caspase-1, and ASC. It seems that the downregulation of cleaved caspase-1 leads to the inhibition of caspase-1 activity by celecoxib (Fig. 3). By contrast, exposure to nimesulide did not result in remarkable alterations in caspase-1 activity in cells (Fig. 4a). This observation was inconsistent with the increase in cleaved caspase-1 expression level induced by nimesulide (Fig. 3).

### Effects of celecoxib and nimesulide on IL-1β secretion

**Fig. 5:**
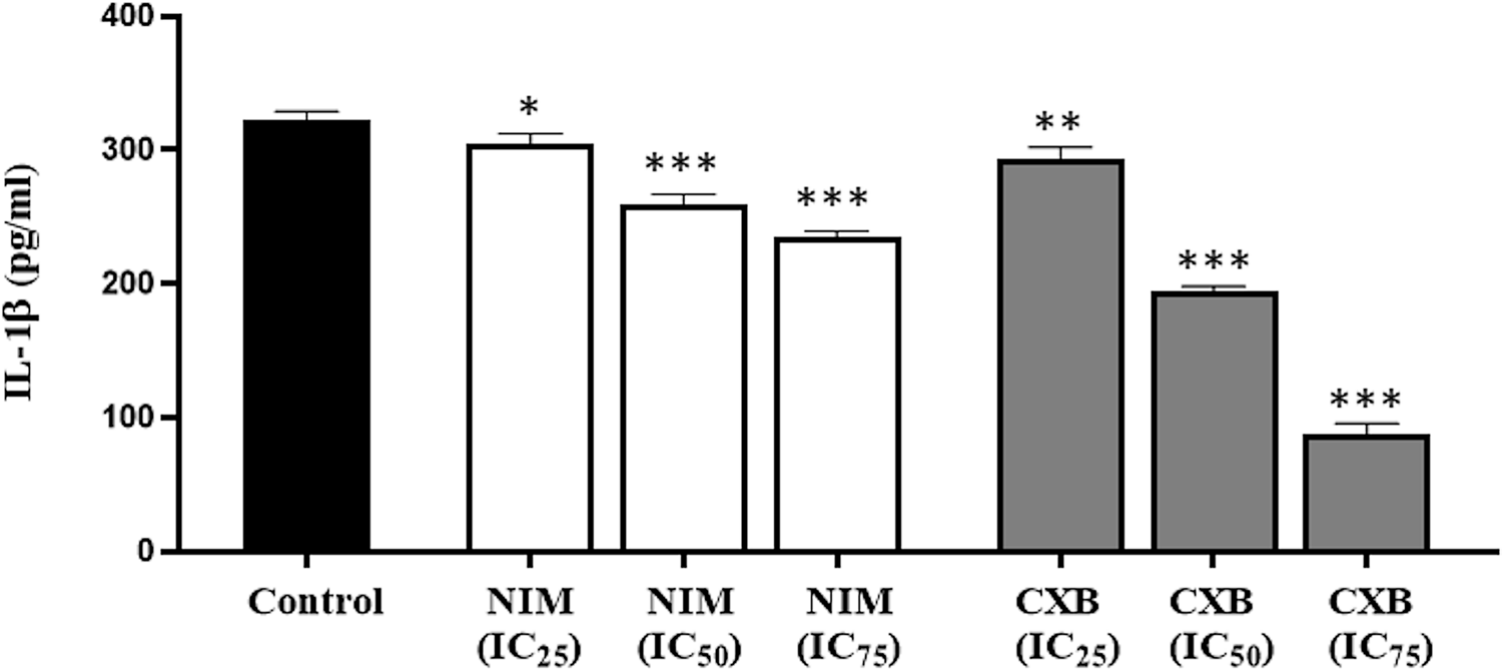
The effects of celecoxib and nimesulide on the levels of IL-1β. MDA-MB-231 cells were treated with different concentrations of each drug for 48 h. The levels of IL-1β were measured in the cell supernatant by an ELISA assay. NIM, nimesulide; CXB, celecoxib. 0.1% DMSO served as a control. Values were represented as mean ± S.D. *p < 0.05, **p < 0.001, and compared to ***p < 0.0001, compared to control

Figure 5 represents the levels of IL-1β in untreated or treated cell supernatants. Both drugs have remarkably decreased the release of IL-1β. The concentrations of IL-1β were reduced by 19.7% and 39.5% with celecoxib and nimesulide, respectively, at their IC_50_ concentrations (Fig. 5).

Importantly, we also observed that celecoxib decreased the protein levels of NLRP1 inflammasome components and activity of caspase-1 (Figs. 3 and 4). All results together suggest that celecoxib inhibits the activation of NLRP1 inflammasome, which may play a role in its anti-proliferative effects. However, there was no clear relationship between decreased IL-1β levels, unchanged caspase-1 activity, upregulation of NLRP1, and cleaved caspase-1 proteins (Figs. 3 and 4).

## Discussion

Inflammation has emerged as a substantial target of cancer treatment and prevention due to its tumor-promoting effect (Landskron et al. 2014). Cancer cells may use specific components of the inflammatory pathways to prevent apoptosis and promote proliferation, angiogenesis, and metastasis. The pro-inflammatory cytokines have been demonstrated as signalling molecules contributing to metastasis by promoting breast cancer cells to penetrate blood vessels (Coussens and Werb 2002). COX-2 and inflammasomes, among various inflammation-related factors, are frequently targeted for anti-inflammatory and anticancer treatments (Alle and Jones 2015; Díaz-Cruz et al. 2005). Celecoxib, a specific COX-2 inhibitor, was proven to exhibit chemopreventive and anticancer activity in several preclinical studies and clinical trials (Basu et al. 2005; Brandão et al. 2013; Bocca et al. 2011; Dai et al. 2012; Woditschka et al. 2008; Lanza-Jacoby et al. 2003). Surprisingly, there are limited studies investigating the anti-antiproliferative effects of nimesulide (Chen et al. 2009; Nakatsugi et al. 2000; Shaik et al. 2004). In our study, celecoxib and nimesulide were found to inhibit the cell viability of MDA-MB-231 in a concentration-dependent manner after 48-h exposure, as expected (Fig. 1). In addition, the colony-forming ability of MDA-MB-231 cells was significantly reduced after treatment with increasing doses of each drug (Fig. 2). We included nimesulide in our study to compare the results of two drugs with different COX-2 inhibition selectivity.

In normal breast tissue, COX-2 is undetectable. However, it is overexpressed by approximately 40% in tumor tissue (Bosco et al. 2011). The overexpression of COX-2 increases by 80% in ductal carcinoma in situ (Díaz-Cruz et al. 2005). The elevated expression level of COX-2 is associated with tumor size, malignancy, and metastasis to lymph nodes and lungs (Díaz-Cruz et al. 2005; Liu et al. 2001). Hence, targeting COX-2 and studying the potential of COX-2 inhibitors for breast cancer therapy is a promising strategy. Bocca et al. demonstrated that celecoxib led to inadequate inhibition of COX-2 in MCF-7 (ER +) cells while inducing a significant antiproliferative effect through ERK/AKT and other contributing mechanisms (Bocca et al. 2011). However, in MDA-MB-231 cells (triple negative), strong suppression of COX-2 was shown to be associated with inhibition of cell growth. Numerous studies indicate that COX-2 expression is higher in MDA-MB-231 cells than in less invasive cells (Basu et al. 2005). Different mechanisms may play a role in the antiproliferative effects of celecoxib depending on the cell line due to invasiveness and COX-2 levels (Li et al. 2018). Therefore, we restricted our investigation to studying only with a triple-negative cell line. Consistent with previous studies, we demonstrated that both drugs exhibit selective and significant cytotoxicity in MDA-MB-231 cells (Fig. 1) and almost wholly inhibit COX-2 activity at the dose levels we studied. The IC_50_ values for inhibition of COX-2 activity were 0.343 and 0.56 µM for celecoxib and nimesulide, respectively (data not shown).

Although the efficacy of celecoxib has been encouraging, the mechanisms of its antitumor action still require investigation. Several mechanisms have been proposed for the antitumor effects of celecoxib, including cytokine-driven upregulation of PGE-synthase-1, regulation of tumor microenvironment, and induction of cell death pathways (Li et al. 2018; Wen et al. 2020). However, there are still questions regarding the exact mechanism underlying its antiproliferative properties (Li et al. 2018). Moreover, the role of NLRP1 inflammasome in the antitumor mechanism of celecoxib is unknown. The current study aimed to demonstrate whether NLRP1 inflammation is involved in the anticancer mechanism of celecoxib and nimesulide in MDA-MB-231 cells.

NLRP1 has been demonstrated to increase the growth of cancer cells by inducing inflammasome activation and suppressing apoptotic pathways (Zhai et al. 2017). Wei et al. reported that NLRP1 was overexpressed in 83% of primary breast cancer tissue. In that study, researchers evaluated the correlation between the expression of NLRP1 and various pathological characteristics of breast cancer patients. NLRP1 protein was indicated to be overexpressed in all human breast cancer subtypes, and a significant association between increased NLRP1 levels and lymph node metastasis was demonstrated. Moreover, it was revealed that NLRP1-overexpressing MCF-7 cells exhibited a high rate of proliferation, migration, and invasion (Wei et al. 2017). Hence, the excessive NLRP1 inflammasome activation may promote tumorigenesis and carcinogenesis in breast tissue. However, cell-based investigations were limited to MCF-7 cells in that study. Therefore, further research is required to explore the NLRP1 expression in MDA-MB-231 cells and its role in the pathogenesis and treatment of triple-negative breast cancer. Our study has demonstrated that NLRP1 protein is slightly expressed in normal breast tissue, while its level was significantly upregulated in MCF-7 cells, as indicated by Wei et al. Surprisingly, the overexpression of NLRP1 was much more pronounced in MDA-MB-231 cells than in MCF-7 cells (Fig. 3b), suggesting that NLRP1 may be a potential therapeutic target in the treatment of triple-negative breast cancer as well as ER ( +) breast cancer.

After demonstrating the expression of NLRP1 protein in three different breast cancer cells and highlighting its dramatic upregulation in MDA-MB-231 cells, we investigated whether COX-2 enzyme inhibitors affect the expression of NLRP1 inflammasomal components in MDA-MB-231 cells. Our results revealed that celecoxib treatment at IC_50_ concentration downregulated NLRP1, pro-caspase-1, and ASC expression in MDA-MB-231 cells (*p* < 0.05) (Fig. 3c). In cancer, excessive NLRP1 activation has been linked to altered protein expression and changes in genes encoding inflammatory pathways (Ciążyńska et al. 2020). Therefore, agents inhibiting mRNA or protein expression of inflammasome components reduce NLRP1 activation and the inflammatory response in the tumor microenvironment. In our study, the decreased protein levels of NLRP1, pro-caspase-1, and ASC induced by celecoxib (IC_50_) may be related to the drug’s cytotoxic effect. Contrary to celecoxib, nimesulide induced the levels of NLRP1, cleaved caspase-1, and ASC in MDA-MB-231 cells (Fig. 3c). This finding was surprising because we expected nimesulide may also downregulate NLRP1, suggesting that celecoxib and nimesulide have different mechanisms of antiproliferative effects on triple-negative breast cancer cells.

Caspase-1 is the main effector protein of NLRP1 inflammasome and is required for the maturation of pro-IL-1β to IL-1β (Franchi et al. 2009). Inflammasome-mediated caspase-1 activation plays a crucial role in regulating the expression of pro-inflammatory genes or apoptotic pathways, making caspase-1 an essential factor in the pathogenesis and treatment of various diseases, including cancer (Lillo and Saleh 2022). However, various developed reagents targeting caspase-1 have limited use due to off-target effects and adverse effects due to extreme prevention of inflammasome activities (Green and Kroemer 2005). In our study, celecoxib inhibited caspase-1 activity in a dose-dependent manner (Fig. 4), which is consistent with a decrease in caspase-1 expression levels (Fig. 3). The activity of caspase-1 remained unchanged with nimesulide treatment, even at IC_75_ concentrations. This data suggests that nimesulide induces the expression of caspase-1 but does not alter its activity.

**Fig. 6:**
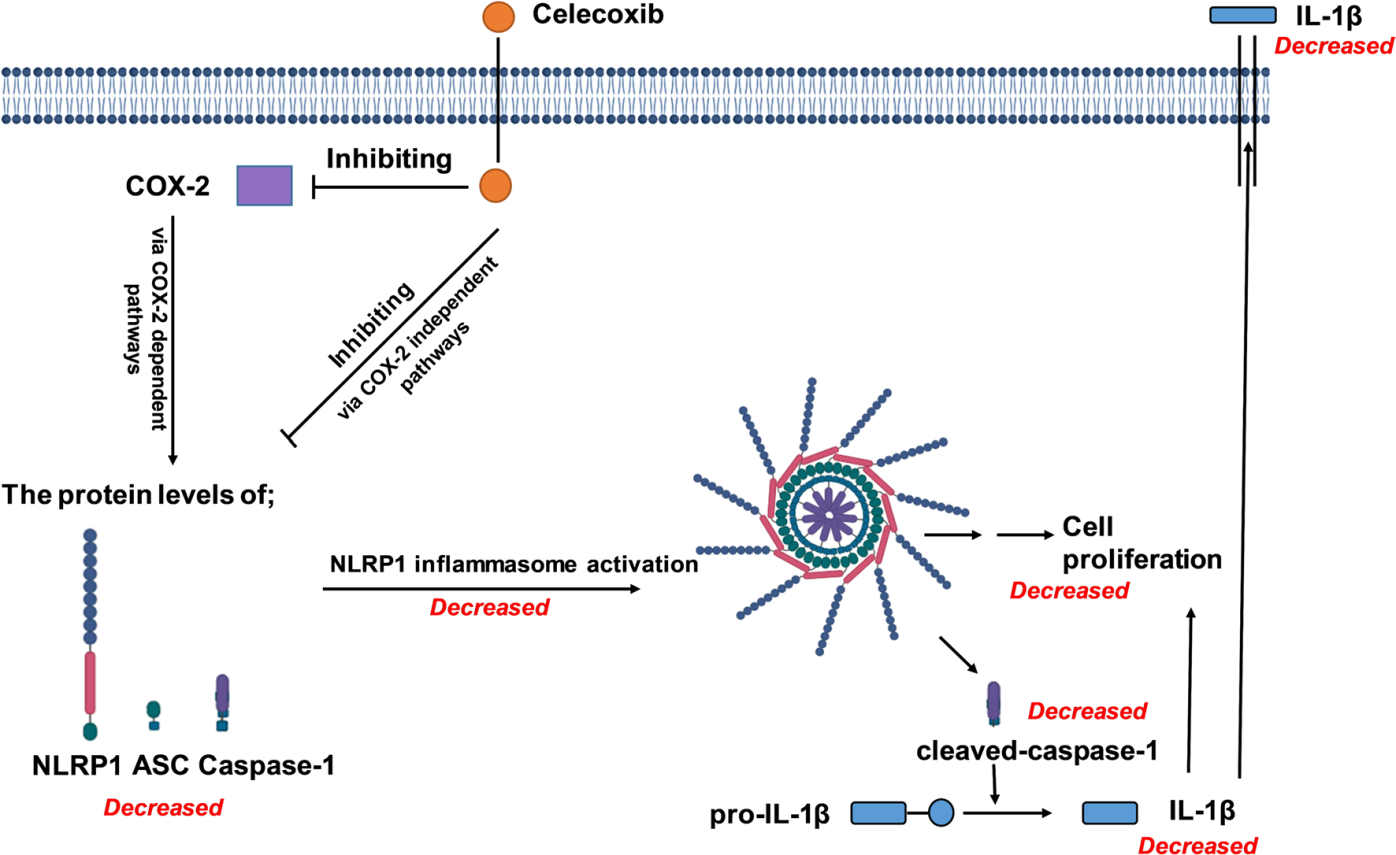
A proposed schematic model presenting the mechanism of antiproliferative effects induced by celecoxib in MDA-MB-231 cells. Exposure to celecoxib downregulates the protein expression of NLRP1, which is markedly upregulated in MDA-MB-231 cells. Moreover, celecoxib inhibits the protein levels of ASC and cleaved caspase-1. The decrease in NLRP1 inflammasome activation resulted in lower levels of caspase-1 activation and IL-1β secretion

Increased levels of the pro-inflammatory cytokine IL-1β in the tumor microenvironment and serum have been associated with tumor growth, progression, angiogenesis, and recurrence in breast cancer (Soria et al. 2011). The IL-1β/IL-1 receptor (IL-1R)/β-catenin pathway increases cancer cell growth and metastasis, leading to higher expression of transcription factors, including SNAIL-1 and c-MYC. In addition, positive feedback in the tumor microenvironment promotes the levels of IL-1β and, unfortunately, carcinogenesis (Escobar et al. 2015). Moreover, it is demonstrated that IL-1β regulates the expression of COX-2, which plays a negative role in mammary lesions (Reed et al. 2009), and inhibition of IL-1/IL1R or knockdown of IL-1R reduced tumor progression. Our study’s final observation was that both drugs decreased IL-1β levels at all investigated doses (Fig. 5). Consistently, the expression levels of NLRP1 inflammasome components and caspase-1 activity were also reduced after the treatment of celecoxib, compared with untreated control, and these reduced levels of NLRP1 components were accompanied by lower IL-1β secretion (Figs. 3, 4 and 5). Our findings strongly indicate that celecoxib significantly inhibits NLRP1 inflammasome activation in MDA-MB-231 cells (Fig. 6). In previous studies, the significance of COX-2-dependent or -independent mechanisms in celecoxib-induced antiproliferative effects has been highlighted (Chen et al. 2009; Li et al. 2018; Nakatsugi et al. 2000; Shaik et al. 2004; Wen et al. 2020). Therefore, in our future studies, we aim to investigate the individual contributions of these two pathways to the inhibition of NLRP1 inflammasome activation, an underlying mechanism of celecoxib-induced anticancer effects. Moreover, knock-down and overexpression techniques are utilized to determine the therapeutic targets of drugs in human cancer cell lines. Therefore, genetically engineered cell lines can be used in our further study to investigate comprehensively whether the NLRP1 protein contributes to the antiproliferative effects of celecoxib in MDA-MB-231 cells. Interestingly, reduced IL-1β secretion induced by nimesulide was associated with neither higher expression of NLRP1 inflammasome components nor increased caspase-1 activity. The present study shows that while suppressing IL-1β may contribute to nimesulide’s antiproliferative effect, it may not be due to the NLRP1 inflammasome pathway.

The low cytotoxicity of celecoxib in healthy cells and its proven anti-tumor properties are promising regarding the repurposing of celecoxib as an anticancer drug. Our study has improved the knowledge of the underlying mechanism behind the anticancer properties of celecoxib. However, it is also crucial not to ignore celecoxib-related cardiotoxicity. Indeed, chronic use of a high dose of celecoxib (800 mg/day) might increase cardiovascular risk (Howes 2007). Therefore, using celecoxib in advanced cancer patients requires careful consideration of benefits and risks. Chen et al. conducted a meta-analysis and suggested that although the use of celecoxib exhibits crucial antitumor effects in cancer treatment, it also increases the risk of cardiovascular toxicity. The incidence of severe cardiovascular side effects was 1.78 times higher in the celecoxib group (Chen et al. 2014). It was reported that celecoxib at 500 mg/kg and higher doses significantly reduces tumor growth and volume in vivo breast cancer models (Li et al. 2018; Woditschka et al. 2008). In our study, the calculated IC_50_ value for celecoxib in MDA-MB-231 cells was 25.3 µM, higher than Cmax, ~ 600–900 ng/ml for 200 mg tablet (FDA 1998). However, further studies are necessary to determine the effective dose for treating breast cancer in humans and to investigate all potential adverse effects. In addition, developing less toxic and more effective drugs may be recommended by modifying the chemical structure of celecoxib, which has proven antitumor activity in preclinical and clinical studies.

## Conclusions

It can be concluded that celecoxib inhibits the activation of the NLRP1 inflammasome by suppressing its component expression levels, inhibiting caspase-1 and IL-1β secretion. The NLRP1 inflammasome signaling pathway may be involved in celecoxib’s anti-proliferative and cytotoxic effects on MDA-MB-231 cells. Further research on different cell lines and in vivo models is needed to comprehensively understand the precise mechanism of celecoxib on breast cancer cells and investigate the novel mechanism proposed in the present study. The observations of the effects of nimesulide on the NLRP1 inflammasome are contradictory. Our results indicated that the inhibition of cell viability and migration induced by nimesulide in breast cancer is not associated with the NLRP1 inflammasome pathway.

## Data Availability

No datasets were generated or analysed during the current study.
