# Correction: Celecoxib inhibits NLRP1 inflammasome pathway in MDA-MB-231 Cells

**DOI:** 10.1007/s00210-024-03348-5

**Published:** 2024-08-06

**Authors:** Ege Arzuk, Derviş Birim, Güliz Armağan

**Affiliations:** 1 Department of Pharmaceutical Toxicology, Faculty of Pharmacy, Ege University, Bornova, 35040 Izmir Turkey; 2 Department of Biochemistry, Faculty of Pharmacy, Ege University, Bornova, Izmir Turkey

**Correction: Naunyn-Schmiedeberg's Archives of Pharmacology**

10.1007/s00210-024-03286-2

The authors regret that Fig. 2 contained an error. In Fig. 2, two colony formation images for NIM (IC_75_) and CXB (IC_75_) were identical. Only an error has been made in the image of NIM (IC_75_). The correct image of NIM (IC_75_) has been replaced with an incorrect one. However, the values in the graph are correct. Therefore, no changes have been made to the graph and the overall results and conclusions are not affected by this change. The corrected Fig. 2 is provided below. The authors apologize for this error.

**Fig. 2:**
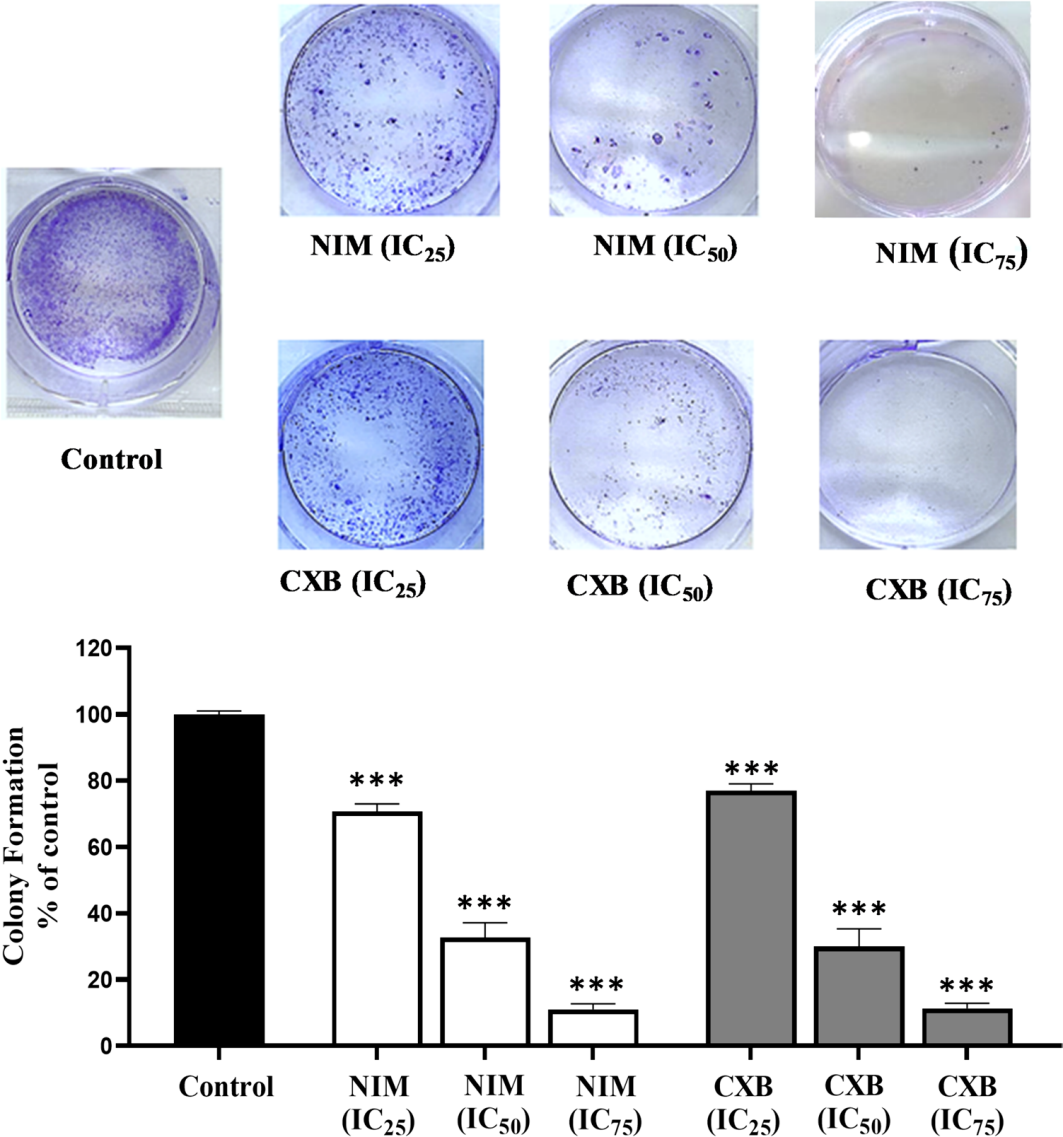
Celecoxib and nimesulide inhibit the colony formation ability of MDAMB-231 cells. Cells were treated with each drug at three dose levels (IC_25_, IC_50_, and IC_75_) for 14 days. Following the treatment period, colonies were stained by 1.25% crystal violet. NIM, nimesulide; CXB, celecoxib. The number of surviving colonies was expressed as a percentage of the control (0.1% DMSO). Values were expressed as mean ± S.D. ***p < 0.0001 (vs. control group)

